# Sporadic Colorectal Cancer Development Shows Rejuvenescence Regarding Epithelial Proliferation and Apoptosis

**DOI:** 10.1371/journal.pone.0074140

**Published:** 2013-10-03

**Authors:** Katalin Leiszter, Orsolya Galamb, Ferenc Sipos, Tibor Krenács, Gábor Veres, Barnabás Wichmann, Alexandra Kalmár, Árpád V. Patai, Kinga Tóth, Gábor Valcz, Béla Molnár, Zsolt Tulassay

**Affiliations:** 1 2nd Department of Internal Medicine, Semmelweis University, Budapest, Hungary; 2 Molecular Medicine Research Unit, Hungarian Academy of Sciences, Budapest, Hungary; 3 1st Department of Pathology and Experimental Cancer Research, Semmelweis University, Budapest, Hungary; 4 1st Department of Pediatrics, Semmelweis University, Budapest, Hungary; Queensland University of Technology, Australia

## Abstract

**Background and Aims:**

Sporadic colorectal cancer (CRC) development is a sequential process showing age-dependency, uncontrolled epithelial proliferation and decreased apoptosis. During juvenile growth cellular proliferation and apoptosis are well balanced, which may be perturbed upon aging. Our aim was to correlate proliferative and apoptotic activities in aging human colonic epithelium and colorectal cancer. We also tested the underlying molecular biology concerning the proliferation- and apoptosis-regulating gene expression alterations.

**Materials and Methods:**

Colorectal biopsies from healthy children (n_1_ = 14), healthy adults (n_2_ = 10), adult adenomas (n_3_ = 10) and CRCs (n_4_ = 10) in adults were tested for Ki-67 immunohistochemistry and TUNEL apoptosis assay. Mitosis- and apoptosis-related gene expression was also studied in healthy children (n_1_ = 6), adult (n_2_ = 41) samples and in CRC (n_3_ = 34) in HGU133plus2.0 microarray platform. Measured alterations were confirmed with RT-PCR both on dependent and independent sample sets (n_1_ = 6, n_2_ = 6, n_3_ = 6).

**Results:**

Mitotic index (MI) was significantly higher (p<0.05) in intact juvenile (MI = 0.33±0.06) and CRC samples (MI = 0.42±0.10) compared to healthy adult samples (MI = 0.15±0.06). In contrast, apoptotic index (AI) was decreased in children (0.13±0.06) and significantly lower in cancer (0.06±0.03) compared to healthy adult samples (0.17±0.05). Eight proliferation- (e.g. MKI67, CCNE1) and 11 apoptosis-associated genes (e.g. TNFSF10, IFI6) had altered mRNA expression both in the course of normal aging and carcinogenesis, mainly inducing proliferation and reducing apoptosis compared to healthy adults. Eight proliferation-associated genes including CCND1, CDK1, CDK6 and 26 apoptosis-regulating genes (e.g. SOCS3) were differently expressed between juvenile and cancer groups mostly supporting the pronounced cell growth in CRC.

**Conclusion:**

Colorectal samples from children and CRC patients can be characterized by similarly increased proliferative and decreased apoptotic activities compared to healthy colonic samples from adults. Therefore, cell kinetic alterations during colorectal cancer development show uncontrolled rejuvenescence as opposed to the controlled cell growth in juvenile colonic epithelium.

## Introduction

The normal cell renewal of the healthy colonic epithelium takes about 5 days, through cell proliferation, differentiation and apoptosis. The location of different types of epithelial cells in the crypts is very typical. Proliferating cells mostly reside on the basis of the crypts, differentiated cells in the middle zone, while the apoptotic cells are close to the luminal surface. Several molecular pathways play important role in the physiological self-renewal of epithelial cells in the human large intestine [1]–[4].

However, the regulation of proliferative and apoptotic mechanisms may alter during normal aging and colorectal carcinogenesis. Aging is a physiologic mechanism that begins after conception. It affects almost every cell in the organism with the possible exception of stem cells and tumor cells. Macroscopic and microscopic changes are present in the aging gastrointestinal tract including the large bowel [5]–[6]. Changes in proliferative activity in the course of aging have been analyzed in animal models in previous studies [7]–[8]. According to the results of *Mandir et al.*, the most intensive epithelial regeneration (proliferation and apoptosis) has been proven in young rats; and the authors concluded that dramatic changes in young colonic epithelium can be related to intestinal development. However, the normal gastrointestinal aging has no affect on epithelial renewal [7]. On the contrary, in aged animal populations increased cell proliferation and decreased apoptosis in colonic epithelium was observed and thought to facilitate uncontrolled cell proliferation and tumorigenesis, which might explain the higher incidence of colorectal cancer in the elderly human [8].

Furthermore, some of these alterations may also be related to colorectal carcinogenesis. Changes in epithelial cell growth and programmed cell death were also previously studied in different stages of colorectal carcinogenesis, but the results are not concordant. Cell proliferation and apoptosis may become dysregulated and the unbalanced cell production and cell loss determine the behavior of premalignant or malignant disorders and tumor growth [9]–[12].

The detection rate of adenomas and the incidence of advanced colorectal adenomas and cancers continually elevate after the age of 40–50, showing strong age dependency [13]–[14]. Sporadic colon cancers turn out mostly in the older adult population similarly to numerous neoplastic and precancerous lesions. According to the Vogelstein model [15], colorectal cancer develops from normal epithelium through pre-malignant adenoma in a multi-step process which takes several years. Besides the genes (e.g. APC, KRAS, DCC and TP53) traditionally implicated in this cancer progression model, recently novel genes with altering mRNA expression have also been suggested to be contribute to malignant transformation of colorectal epithelium [16]–[18].

There are several genetic and epigenetic alterations that can demonstrate a possible relationship between aging and colorectal carcinogenesis. Accumulation of DNA mutations and damages, promoter hypermethylation, alterations in DNA repair, telomerase activity and cellular metabolism may increasingly affect aged populations leading to elevated cell proliferation and decreased apoptosis, which may culminate to malignant transformation and uncontrolled cell proliferation [19].

At the same time, several human and animal studies have revealed the opposing regulation of some molecular pathways during normal aging and carcinogenesis. As opposed to normal aging, proliferating cancer cells show increased metabolism, characterized by continuous proliferative activity and de-differentiation, they can produce embryonic proteins and are potentially immortal by escaping apoptosis [20]. In particular, apoptosis-regulating proteins show distinct expression in senescent and cancer cells featured by the downregulation of the apoptosis-inducing tumor suppressor p53 protein [21] and Fas/CD95 protein [22] and the overexpression of antiapoptotic proto-oncogene Bcl-2 in cancer as opposed to normal aging cells [23]–[25]. Oncogenes such as Ras, transcription factors e.g. Myc, and growth signal transduction-related tyrosine-kinase receptors e.g. members of the EGFR family are up-regulated in some cancers, while downregulated in senescent cells [26]–[28].

Cancer development can be considered as a local, uncontrolled “rejuvenation” utilizing the same molecular pathways but with opposing regulation. Deregulated cell proliferation and apoptosis pathways can allow survival advantages for cancer cells against adjacent senescent cells, due to lost ability of cancer for normal aging [20]. Increased epithelial cell proliferation in the gastrointestinal tract can be seen not only in colorectal cancer, but also in embryonic and juvenile development. An essential pathway that plays fundamental roles both in gut development and in sporadic or familial colorectal cancers is the Wnt/β-catenin signaling [29]–[30].

As we are aware, there is no study focusing on the proliferation and apoptosis regulation in the juvenile human colorectal epithelium in relation to normal aging and carcinogenesis. The purpose of this study was to analyze the proliferative and apoptotic activity in human colonic epithelium in the course of normal aging and colorectal carcinogenesis both at protein and gene expression level. Colorectal biopsies representing the juvenile controlled growth stage, the adult healthy status and the uncontrolled colorectal cancer development were tested for potential correlations.

## Materials and Methods

### Patients and samples

After informed consent, colorectal biopsy samples were taken during routine endoscopic intervention at the 2nd Department of Internal Medicine and 1st Department of Paediatrics, Semmelweis University, Budapest, Hungary. Altogether, 44 tissue samples were analyzed in the immunohistochemical study (14 healthy children, 10 healthy adults, 10 adenomas from adults and 10 CRCs from adults); 81 biopsy samples were analyzed in microarray analysis (6 healthy children, 41 healthy adults and 34 CRCs from adults); and 36 biopsy samples were involved in real-time PCR validation (12 healthy children, 12 healthy adults and 12 CRCs from adults). Seventy-five microarrays (the adult samples) had been hybridized earlier; their data files were used in a previously published studies using different comparisons [31]–[32] and are available in the Gene Expession Omnibus database (series accession number: GSE10714 and GSE37364). The datasets of the newly hybridized 6 microarrays are registered on the GSE37267 serial accession number. Control children were referred to the outpatient clinic with rectal bleeding, constipation or chronic abdominal pain. Ileocolonoscopy was part of their diagnostic procedure (exclude organic disease) and the biopsy specimens showed normal macroscopic appearance and histology. Every specimen was verified by histopathologists.

For immunohistochemistry colorectal biopsy samples were routinely fixed in formaldehyde and embedded in paraffin wax. For mRNA studies, colonoscopy samples were stored in RNALater Reagent (Qiagen Inc, Germantown, US) at −80°C before total RNA extraction for gene expression analysis and Taqman RT-PCR study. Ethical approvals (Nr.: 69/2008 and 202/2009) for this study were issued by the Regional and Institutional Committee of Science and Research Ethics of Semmelweis University (Budapest, Hungary). Written informed consent was obtained in advance from all adult participants and from the next of kin, caretakers, or guardians on the behalf of the minors/children approved by the ethics committees. Detailed clinicopathological specification of the patient samples are summarized in *Table 1* and *Table 2*.

**Table 1:** Subgroups of patients participating in immunohistochemistry, microarray analysis and PCR validation with the number of samples and mean age values.

Number of patients participating in the study		
*Immunohistochemistry*		
Group	Number of samples (female/male)	Mean age ± SD (years)
Children (Ch)	14 (7/7)	11.2±5.5
Healthy adults (N)	10 (4/6)	60±14.2
Adenoma from adults (Ad)	10 (4/6)	66.7±3.9
CRCs from adults (CRC)	10 (3/7)	68.5±14.4
**Total patient numbers**	**44 (18/26)**	

*Affymetrix microarray analysis*		
Group	Number of samples (female/male)	Mean age ± SD (years)
Children (Ch)	6 (2/4)	11.2±5.3
Healthy adults (N)	41 (26/15)	53±15.9
CRCs from adults (CRC)	34 (19/15)	68.5±10.3
**Total patient numbers**	**81 (47/34)**	

*Taqman RT-PCR validation*		
Group	Number of original samples (female/male)	Mean age ± SD (years)
Children (Ch)	6 (2/4)	12.2±5.3
Healthy adults (N)	6 (3/3)	57.7±18.9
CRCs from adults (CRC)	6 (4/2)	69±7.0
**Total patient numbers**	**18 (9/9)**	

Group	Number of independent samples (female/male)	Mean age ± SD (years)
Children (Ch)	6 (3/3)	5.5±3.4
Healthy adults (N)	6 (3/3)	53±16.9
CRCs from adults (CRC)	6 (1/5)	64.3±10.8
**Total patient numbers**	**18 (7/11)**	

**Table 2:** Clinical features of healthy children controls participating in immunohistochemistry, microarray analysis and PCR validation.

*Immunohistochemistry*				
Patient number	Signs/Symptoms	Colonoscopy	Histology	Diagnosis
**1.**	Abdominal pain	Negative	Negative	WD
**2.**	Hematochezia	Negative	Negative	WD
**3.**	Hematochezia	Negative	Negative	Constipation
**4.**	Abdominal pain	Negative	Negative	IBS
**5.**	Hematochezia	Negative	Negative	WD
**6.**	Abdominal pain	Negative	Negative	IBS
**7.**	Abdominal pain	Negative	Negative	IBS
**8.**	Chronic diarrhea	Negative	Negative	IBS
**9.**	Hematochezia	Negative	Negative	WD
**10.**	Chronic diarrhea	Negative	Negative	IBS
**11.**	Chronic diarrhea	Negative	Negative	IBS
**12.**	Hematochezia	Negative	Negative	Constipation
**13.**	Abdominal pain	Negative	Negative	WD
**14.**	Hematochezia	Negative	Negative	Constipation

*Affymetrix microarray analysis/Taqman RT-PCR validation - original samples*				
Patient number	Signs/Symptoms	Colonoscopy	Histology	Diagnosis
**1.**	Hematochezia	Negative	Negative	WD
**2.**	Hematochezia	Negative	Negative	WD
**3.**	Abdominal pain	Negative	Negative	WD
**4.**	Vomiting	Negative	Negative	IBS
**5.**	Abdominal pain	Negative	Negative	IBS
**6.**	Abdominal pain	Negative	Negative	WD

*Taqman RT-PCR validation - independent samples*				
Patient number	Signs/Symptoms	Colonoscopy	Histology	Diagnosis
**1.**	Hematochezia	Negative	Negative	WD
**2.**	Hematochezia	Negative	Negative	WD
**3.**	Abdominal pain	Negative	Negative	IBS
**4.**	Hematochezia	Negative	Negative	WD
**5.**	Constipation	Negative	Negative	Constipation
**6.**	Hematochezia	Negative	Negative	IBS

WD = Without disease, IBS = Irritable bowel syndrome.

### Immunohistochemistry for cell proliferation

Archived samples from 14 histologically normal colonic biopsies from children, 10 normal colonic biopsies from adults, as well as 10 adult adenomas and 10 CRCs were collected during routine endoscopy. 1 mm-diameter cores were punched out from paraffin blocks of adult samples and collected into tissue microarrays (TMA). Four µm thick sections cut both from the TMAs and from individual children biopsy samples were dewaxed and rehydrated for immunostaining.

After antigen retrieval in a pH 9.0 TRIS-EDTA buffer using a microwave oven at 900 W for 10 min and then at 370 W for 40 min, blocking of endogenous peroxidases in 1% hydrogen peroxide dissolved in methanol and of nonspecific binding sites using 1% bovine serum albumin for 20 min each were performed. Slides were incubated with mouse monoclonal anti-Ki-67 antibody (Clone: MIB-1, 1∶100, Dako, Glostrup, Denmark) for 60 min in a humidified chamber and then with anti-mouse IgG F(ab′)_2_ Alexa Fluor 546 conjugate (1∶200, Invitrogen, Carlsbad, CA, USA) for 30 min.

### Apoptosis detection using the Tdt-mediated dUTP Nick End Labeling (TUNEL) assay

After digestion with proteinase-K (20 µg/ml, 20 min), 50 µl TUNEL (TUNEL In Situ Cell Death Detection Kit, Fluorescein, Roche, 11684795910) reaction mixture (5 µl TdT enzyme+45 µl dUTP) was added to the tissue sections and TMA slides. Then the samples were incubated in a dark humidified chamber at 37 C° for 120 minutes. Cell nuclei were stained with Hoechst (5 µl Hoechst dye+10 ml TBS, 1 min, Sigma-Aldrich, St. Louis, Mo, USA).

### Counting of mitotic and apoptotic index in digital slides

Stained biopsy samples and TMA slides were digitalized with a high resolution digital scanner (Pannoramic Scan, 3DHISTECH Ltd. Budapest, Hungary) using multilayer fluorescent scanning with a high numeric aperture (0.8)×20 objective lens and a high dynamic range AxioCam Mrm Rev.3 black-and-white camera connected to the scanner. Digital slides were accessed through a computer monitor and analyzed using the Pannoramic Viewer software (version 1.11.43.0). The Marker Counter software module resulting in permanent annotations on the counted cells was used to estimate the relative ratio of proliferative, apoptotic and normal cells.

Ki-67, TUNEL and Hoechst positivities appeared all as strong nuclear labeling in the slides. Depending on sample size, 500–1000 epithelial cells were counted in longitudinal well-oriented crypts. We have determined the proliferative-apoptotic ratio (PAR: ratio of proliferative and apoptotic cells in crypts), the mitotic index (MI: the ratio of proliferative cells and total counted cells in crypts) and the apoptotic index (AI: the ratio of apoptotic cells and total counted cells in crypts).

### mRNA microarray expression analysis

Total RNA was extracted using RNeasy Mini Kit (Qiagen Inc., Germantown, USA) according to the manufacturer's instructions. Quantity of isolated RNA was characterized by measuring absorbance (NanoDrop ND-1000 Spectrophotometer, NanoDrop Technologies, Inc., Wilmington, USA). Quality of isolated RNA was tested with capillary gel electrophoresis (2100Bioanalyzer and RNA 6000 Pico Kit/Agilent Inc., Santa Clara, CA, USA/). Biotinylated cRNA probes were synthesized from 1 to 8 µg total RNA and fragmented using the One-Cycle Target Labeling and Control Kit (http://www.affymetrix.com/support/downloads/manuals/expression_s2_manual.pdf), according to the Affymetrix description. Twenty micrograms of each fragmented cRNA sample was hybridized into HGU133 Plus2.0 array (Affymetrix) at 45°C for 16 h. Slides were washed and stained using Fluidics Station 450 and antibody amplification staining method according to the manufacturer's instructions. Fluorescent signals were detected by GeneChip Scanner 3000 (Affymetrix).

### TaqMan RT-PCR

After microarray gene expression analysis, results were compared with other data set available in the Gene Expression Omnibus databank (http://www.ncbi.nlm.nih.gov/geo; series accession number is GSE18105). TaqMan polymerase chain reaction was used to measure mRNA expression of 12 selected genes, participating in regulation of cell cycle, proliferation or apoptosis, on original (6 histologically intact children, 6 histologically intact adult and 6 CRC adult samples) and independent sets of samples (6 histologically intact children, 6 histologically intact adult, 6 CRC adult samples). 18S ribosomal RNA and GAPDH were used as reference.

Total RNA extraction, quality and quantity controls were performed as described earlier. Using the High Capacity cDNA Reverse Transcription Kit with RNase Inhibitor, 1 µg of total RNA per sample was reverse transcribed (Life Technologies, Carlsbad, CA, USA). The quality of cDNA was checked by SDHA real-time PCR (F. Hoffmann-La Roche Ltd., Basel, Switzerland). Then 16.7 ng cDNA template per sample was used for expression analysis of selected genes with TaqMan Gene Expression Master Mix (Life Technologies). The measurement was carried out on LightCycler 480 (Roche) with Mono Colour Hydrolysis Probe/UPL Probe detection format. After denaturation at 95°C for 10 min, 40 PCR cycles were carried out (amplification at 95°C for 15 sec, and at 60°C for 1 min).

### Statistical evaluation

For statistical analysis of Ki-67 immunostaining and TUNEL results the ANOVA test and Tukey HSD post-test were applied. In both methods significance criteria was p<0.05.

For mRNA expression profiles the Affymetrix expression arrays were primarily pre-processed by GCRMA background correction method with quantile normalization and median polish summarization. SAM analysis was applied for determination of proliferation- and apoptosis-regulating genes with altering mRNA expression. The following SAM criteria were used: LogFC≥abs 1, p-value<0.05. All of these genes were compared and the differentially expressed ones were further investigated. The expression of each selected gene among different sample groups was further analyzed by ANOVA and post-test Tukey HSD.

The datasets are available in the Gene Expression Omnibus databank (http://www.ncbi.nlm.nih.gov/geo/), series accession numbers: GSE10714, GSE37364 and GSE37267.

For real-time PCR validation 12 samples were analyzed in each stage (children, healthy/normal adults and adult CRCs). For normalization, 18S ribosomal RNA was used as internal control. For statistical analysis ANOVA test and Tukey HSD post-test were applied. The following criteria were used: Fold change≤0.5 or Fold change≥2 and p-value<0.05.

## Results

### Proliferation and apoptosis in juvenile, normal adult, adenoma and colorectal carcinoma samples

**Figure 1:**
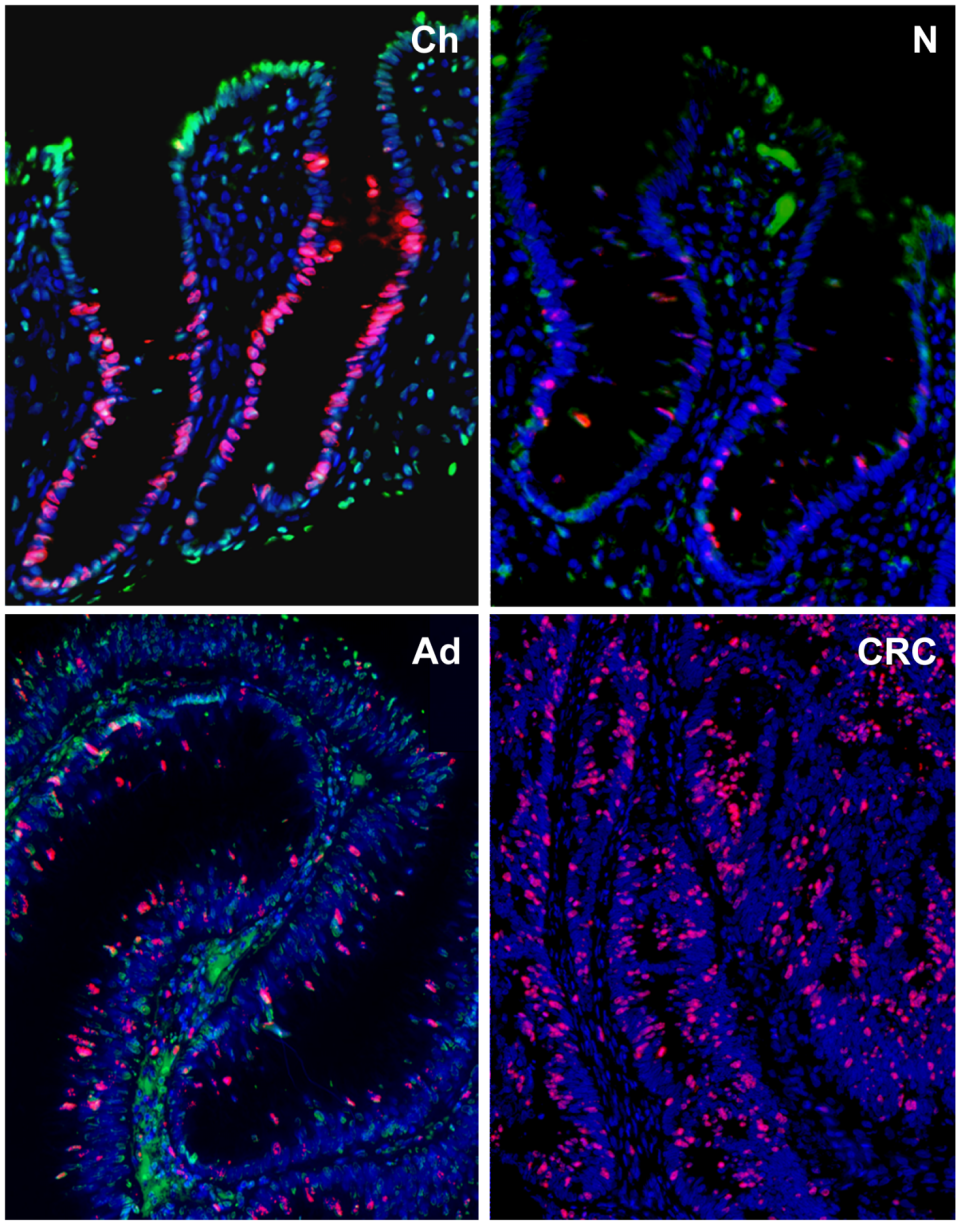
Detection of proliferative (red nuclei) and apoptotic (green nuclei) cells during aging and colorectal adenoma-carcinoma sequence (ACS) with fluorescent staining. Blue spots represent the nuclei of inactive cells. Images were taken with digital microscope: normal child tissue (Ch), normal adult tissue (N), adenoma (Ad) and carcinoma (CRC) in adult. Mitotic activity decreases during aging and increases during the ACS in contrast to apoptotic activity.

**Figure 2:**
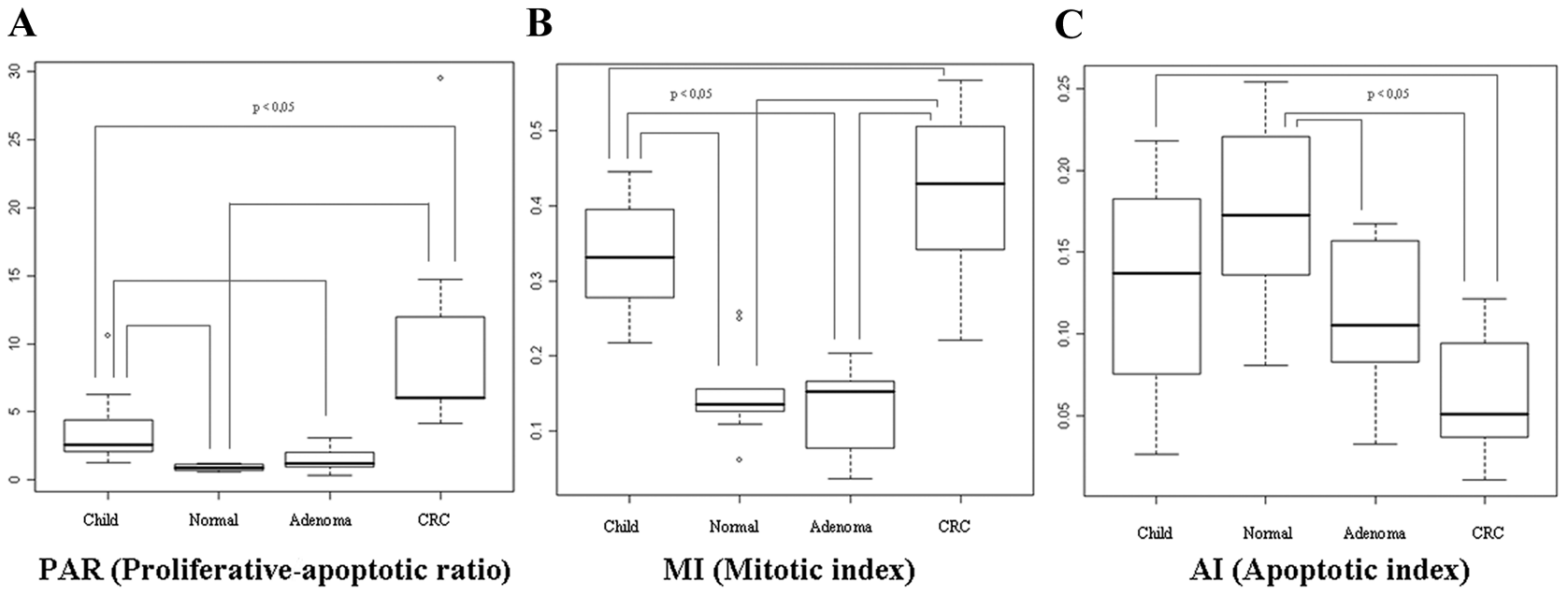
Alterations of proliferative-apoptotic ratio /PAR/ (A), mitotic index /MI/ (B) and apoptotic index /AI/ (C) during aging in histologically intact colonic mucosa and in ACS. PAR and MI decrease during aging and increase during carcinogenesis, contrary to AI.

Proliferating epithelial cells were predominantly localized at the base of crypts while apoptotic cells were close to the luminal surface in normal colonic mucosa *(* *Figure 1* *)*. Proliferative-apoptotic ratio (PAR: ratio of proliferative and apoptotic epithelial cells in crypts) and MI were significantly higher in children (PAR = 3.51±2.49; MI = 0.33±0.06) and CRC samples (PAR = 9.83±7.72; MI = 0.42±0.10) than in healthy adult samples (PAR = 0.88±0.22; MI = 0.15±0.06) (p<0.05). They showed continuous increase in the course of adenoma-carcinoma sequence (ACS). PAR was 3.99 times higher in healthy children colonic epithelium and 11.17 times higher in CRC as compared to healthy adult epithelium; and PAR was 2.8 times higher in CRC in contrast to the juvenile samples. Based on our immunhistochemical results, MI was 2.2 times higher in children and 2.8 times higher in CRC compared that to healthy normal, and 1.27 times higher in CRC in contrast to juvenile samples. The highest PAR and MI were found in colorectal cancer samples *(* *Figure 2* *)*.

AI was decreased in healthy children samples (0.13±0.06) and significantly lower in colorectal cancer samples (0.06±0.03) than in the histologically intact adult colonic samples (0.17±0.05) (p<0.05). AI showed continuous decrease in parallel with the colorectal ACS. Apoptotic index was proven 0.76 times lower in children, and 0.35 times lower in CRC compared to healthy normal samples; and it was 0.46 times lower in CRC comparing to juvenile samples. The lowest AI was detected in colorectal cancer samples *(* *Figure 1* *–* *2* *)*.

Significant alteration in proliferative activity was not found between healthy adult colonic mucosa (PAR = 0.88±0.22; MI = 0.15±0.06) and adenoma samples (PAR = 1.45±0.89; MI = 0.13±0.05); while the AI was found to be significantly lower in adenoma samples (Normal AI = 0.17±0.05; Adenoma AI = 0.10±0.04) (p<0.05).

### Expression of proliferation- and apoptosis-regulating genes in juvenile, normal adult and colorectal carcinoma samples

**Figure 3:**
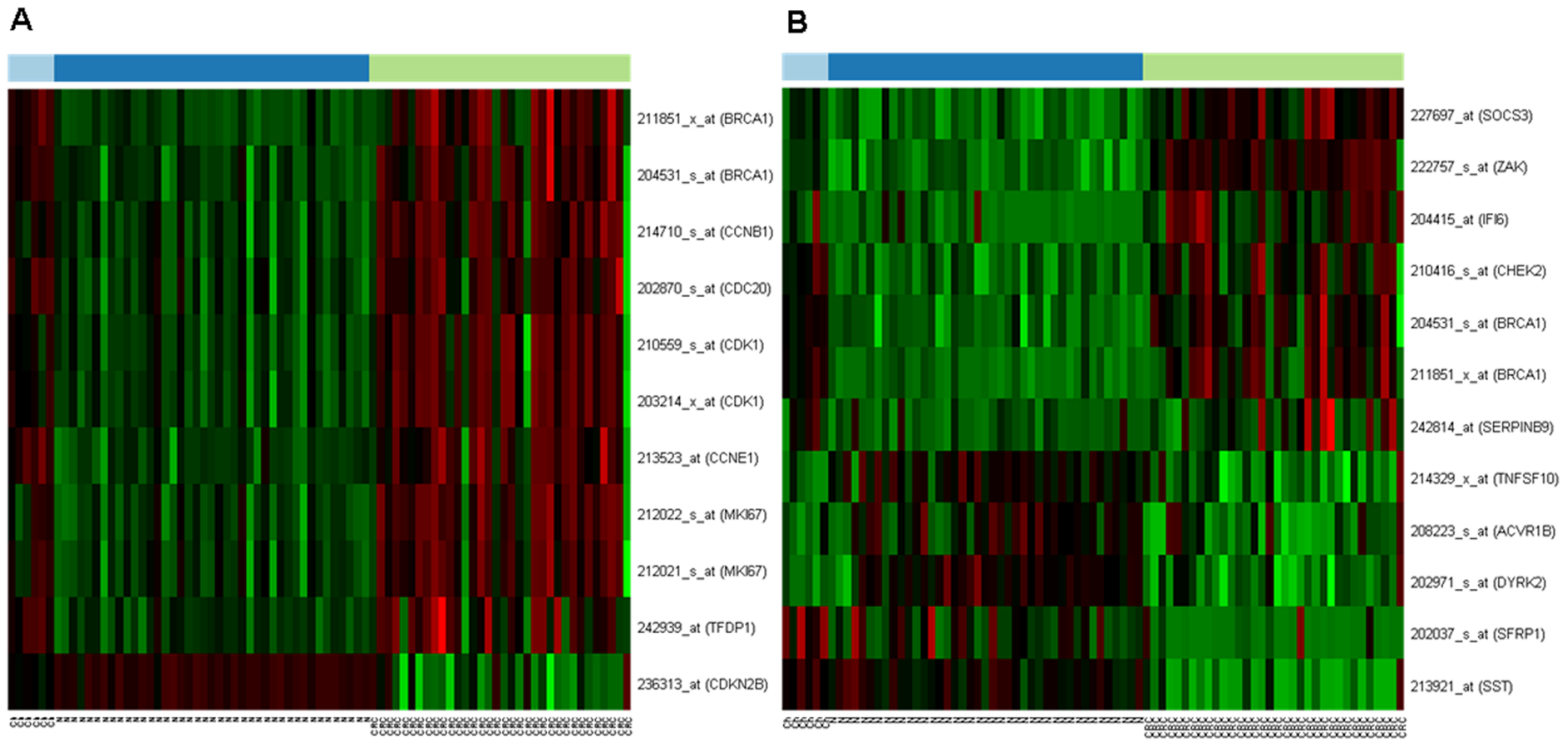
Changes in mRNA expression of proliferation- (A) and apoptosis-regulating genes (B) during aging (Children /Ch/ vs. Healthy adult /N/ colonic epithelium) and carcinogenesis (Healthy adult /N/ vs. Colorectal cancer /CRC/) using Affymetrix HGU133 Plus2.0 array. On the heat map, increased gene expression is represented with red lines, while decreased expression with green. The first 6 samples with light blue show genes in children, the dark blue are from healthy adults, while the last samples in green are from cancer patients.

mRNA expression of 117 proliferation-regulating genes were studied in HGU133 Plus2.0 microarrays. Gene expression of 5 probes (belonging to 4 genes) altered in the course of aging alone in histologically intact colonic mucosa; mRNA expression of 18 probes (belonging to 13 genes) were altered during colorectal carcinogenesis; and 11 probes (belonging to 8 genes /BRCA1, CCNB1, CCNE1, CDC20, CDK1, CDKN2B, MKI67 and TFDP1/) were differently expressed in both group of samples (*Figure 3A*). Similarly, gene expression of 534 apoptosis-regulating genes was also analyzed in this study. mRNA expression of 15 probes (belonging to 9 genes) altered in the course of aging alone in histologically intact colonic mucosa; 46 probes (belonging to 32 genes) showed changes during colorectal carcinogenesis; and 12 probes (belonging to 11 genes /ACVR1B, BRCA1, CHEK2, DYRK2, IFI6, SERPINB9, SFRP1, SOCS3, SST, TNFSF10 and ZAK/) were differently expressed in both group of samples *(* *Figure 3B* *)*.

### Gene expression alterations between juvenile and CRC samples

**Figure 4:**
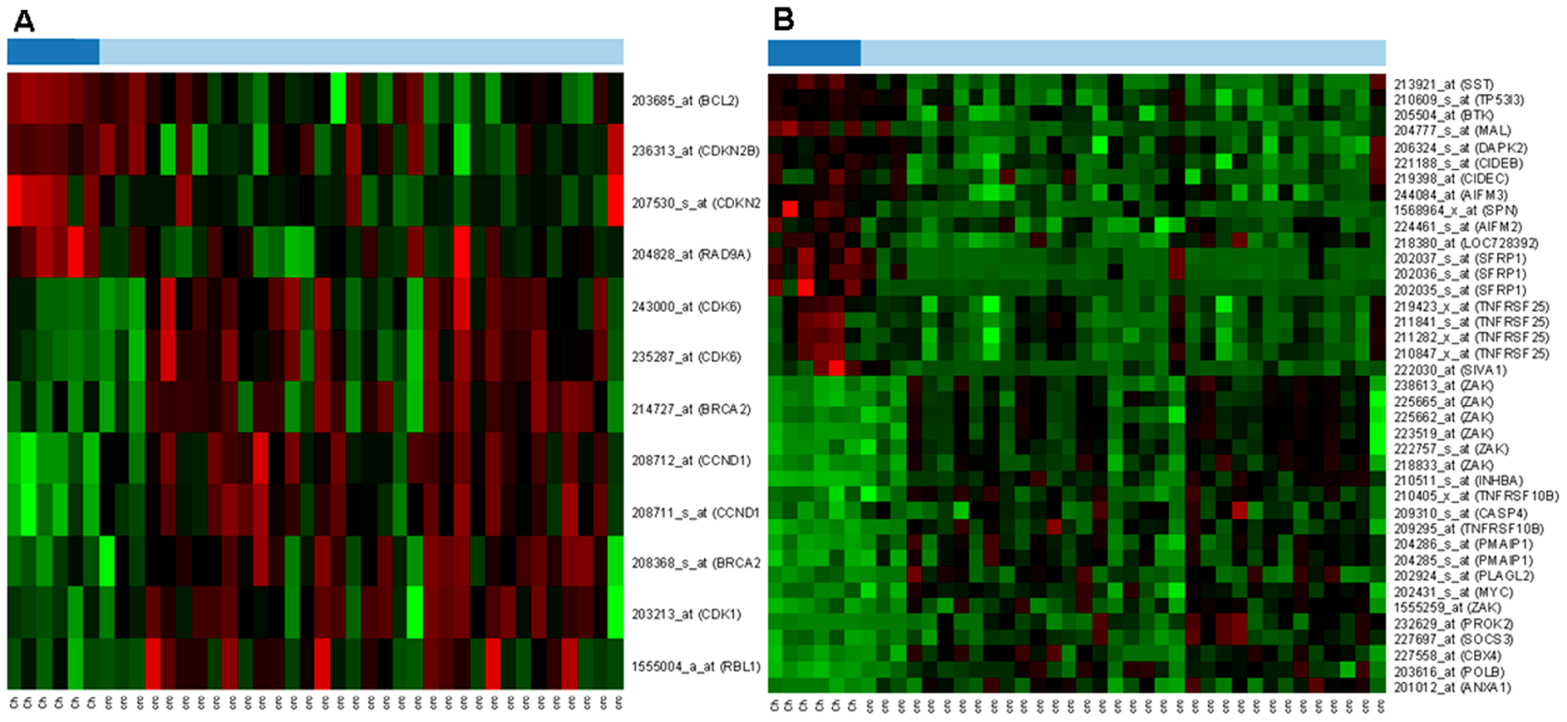
Proliferation (A) and apoptosis (B) controlling genes, that showed significant mRNA expression alterations between healthy young (Ch) and colorectal cancer (CRC) samples. On the heat map, increased gene expression is represented with red lines, while decreased expression with green. The dark blue samples are from healthy children colonic mucosa (Ch) and the light blue are from colorectal cancer samples (CRC).

Proliferation- and apoptosis-regulating genes were further investigated to find genes with dissimilar mRNA expression that can explain the differences between the controlled and uncontrolled cellular proliferation. Twelve probes belonging to 8 proliferation-controlling genes (BCL2, CDKN2B, RAD9A, BRCA2, CCND1, CDK1, CDK6 and RBL1) *(* *Figure 4A* *)* and 26 apoptosis-regulating genes (AIFM2, AIFM3, BTK, CIDEB, CIDEC, DAPK2, MAL, NLRP1 (LOC728392), SFRP1, SIVA1, SPN, SST, TR53I3, TNFRSF25, ANXA1, CBX4, CASP4, INHBA, MYC, PLAGL2, PMAIP1, POLB, PROK2, SOCS3, TNFRSF10B and ZAK) with 39 probes *(* *Figure 4B* *)* showed significant alteration between children and cancer groups, according to the p-value.

### Validation of gene expression using TaqMan RT-PCR

mRNA expression of 10 genes including CDKN2B, MKI67, CDC2/CDK1, CCNE1, ACVR1B, TNFSF10, DYRK2, SOCS3, IFI6 and SERPINB9 were validated with TaqMan RT-PCR *(* *Table 3* *)*.

**Table 3:** Cell proliferation- and apoptosis-regulating genes analyzed in the study.

Gene symbol	Affymetrix ID	Taqman ID	Gene name	Gene function
**CDKN2B**	236313_at	Hs00365249_m1*	Cyclin-dependent kinase inhibitor 2B (p15, inhibits CDK4)	Negative regulation of (epithelial) cell proliferation.
**MKI67**	212022_s_at	Hs01032443_m1*	Antigen identified by monoclonal antibody Ki-67	Cell proliferation/cell cycle regulation.
**CDC2/CDK1**	210559_s_at	Hs00364293_m1	Cell division cycle 2, G1 to S and G2 to M	Cell cycle and cell division regulation. Anti-apoptosis.
**CCNE1**	213523_at	Hs01026536_m1*	Cyclin E1	Cell cycle and cell division regulation. Organ development and regeneration.
**ACVR1B**	208223_s_at	Hs00923304_m1	Activin A receptor, type IB	Induction of apoptosis.
**TNFSF10**	214329_x_at	Hs00234356_m1*	Tumor necrosis factor (ligand) superfamily, member 10	Induction of apoptosis.
**DYRK2**	202971_s_at	Hs00705109_s1*	Dual-specificity tyrosine-(Y)-phosphorylation regulated kinase 2	Induction of apoptosis.
**SOCS3**	227697_at	Hs02330328_s1*	Suppressor of cytokine signaling 3	Anti-apoptosis. Regulation of growth. Response to hypoxia. Aging.
**IFI6**	204415_at	Hs00242571_m1*	Interferon, alpha-inducible protein 6	Anti-apoptosis.
**SERPINB9**	242814_at	Hs00244603_m1*	Serpin peptidase inhibitor, clade B (ovalbumin), member 9	Anti-apoptosis.

According to the results of microarray analysis of proliferation-regulating genes (CDKN2B, MKI67, CDC2/CDK1 and CCNE1), significant mRNA expression alterations were detected by the value of Fold changes (FC≤0.5 or FC≥2) and the ANOVA-test (p<0.05) in Children vs. Normal and Normal vs. CRC comparisons; and these results were also confirmed with Tukey-test in case of CDC2/CDK1 and CCNE1. PCR validation confirmed the tendency of gene expression alterations in all cases with respect to proliferation regulation. CDKN2B, MKI67, CDC2/CDK1 and CCNE1 showed borderline significant mRNA expression changes in previously mentioned comparisons, according to Fold change. Tukey post-test recruited gene expression alterations during aging and colorectal carcinogenesis in case of CDC2/CDK1 (p<0.05).

Numbers of apoptosis-regulating genes (ACVR1B, TNFSF10, DYRK2, SOCS3, IFI6 and SERPINB9) were also analyzed with RT-PCR. Gene expression of ACVR1B, TNFSF10 and DYRK2 was significantly lower in children and CRC samples compared to normal adult colonic mucosa (FC≤0.5 or FC≥2; p<0.05); and these results were validated by RT-PCR as well. According to the results of Affymetrix study, mRNA expression of anti-apoptotic genes, such as SOCS3, IFI6 and SERPINB9, showed significantly higher expression in children and CRC samples as compared that to histologically intact adult colonic samples. PCR validation confirmed the tendency of gene expression alterations between Children vs. Adult Normal and Adult Normal vs. CRC. ANOVA and Tukey-test analysis of RT-PCR results have verified these alterations in case of SOCS3 and IFI6 (p<0.05). Expression changes of the selected genes are summarized in *Table 4*.

**Table 4:** Averages and standard deviation of normalized log2 intensities on microarray and RT-PCR, with ANOVA analysis.

Gene symbol	Affymetrix ID	Averages and standard deviations of normalized log2 intensities on microarrays			ANOVA on microarray
		Children (Ch)	Healthy adults (N)	CRCs (CRC)	
**CDKN2B**	**236313_at**	10.13±0.45	11.64±0.61	7.74±2.55	**2.90E-14**
**MKI67**	**212022_s_at**	9.02±0.95	7.94±0.78	9.97±1.35	**3.67E-11**
**CDC2/CDK1**	**210559_s_at**	8.73±0.65	7.61±0.69	9.46±1.45	**1.09E-09**
**CCNE1**	**213523_at**	6.89±0.39	5.71±0.4	6.74±0.84	**4.96E-10**
**ACVR1B**	**208223_s_at**	4.75±0.65	5.79±0.8	4.72±1.09	**1.08E-05**
**TNFSF10**	**214329_x_at**	9.87±0.47	10.97±0.52	9.89±0.88	**6.05E-09**
**DYRK2**	**202971_s_at**	10.27±0.37	11.39±0.58	10.24±0.78	**2.12E-10**
**SOCS3**	**227697_at**	8.2±1.04	6.44±1.21	10.27±1.86	**2.20E-16**
**IFI6**	**204415_at**	7.07±1.66	5.92±0.95	7.97±1.49	**4.18E-09**
**SERPINB9**	**242814_at**	5.43±1.36	4.3±0.78	5.39±1.82	**2.20E-03**

Gene symbol	Taqman ID	Averages and standard deviations of normalized intensities in RT-PCR			ANOVA on RT-PCR
		Children (Ch)	Healthy adults (N)	CRCs (CRC)	
**CDKN2B**	Hs00365249_m1*	14.13±0.37	14.37±0.22	13.96±0.44	2.19E-01
**MKI67**	Hs01032443_m1*	22.94±0.51	22.24±0.58	22.63±0.55	1.10E-01
**CDC2/CDK1**	**Hs00364293_m1**	19.73±0.6	18.5±0.82	19.83±1.1	**4.99E-03**
**CCNE1**	Hs01026536_m1*	20.01±0.41	19.43±0.51	19.98±0.8	7.05E-02
**ACVR1B**	**Hs00923304_m1**	15.5±0.93	15.45±1.02	16.08±2.52	4.90E-01
**TNFSF10**	Hs00234356_m1*	22.4±0.26	23.22±0.22	22.55±0.77	**3.22E-03**
**DYRK2**	Hs00705109_s1*	22.82±0.3	23.31±0.37	22.66±0.67	**8.24E-03**
**SOCS3**	Hs02330328_s1*	21.85±1.09	20.29±0.55	22.72±1.41	**1.90E-04**
**IFI6**	Hs00242571_m1*	21.35±1.38	20.42±0.52	22.37±1.23	**8.94E-05**
**SERPINB9**	Hs00244603_m1*	20.57±0.47	20±0.42	20.61±0.75	2.30E-01

P-values of ANOVA analysis on microarray and RT-PCR represents in the last column. The significant different expression (p<0.05) is marked in bold.

## Discussion

Aging is associated with increased incidence of sporadic colorectal malignancies, which is one of the leading causes of mortality in Western countries [33]. Colorectal cancer is related to uncontrolled cellular proliferation and dysregulated apoptosis. Juvenile growth, on the other hand, is characterized by controlled growth, cellular proliferation and apoptosis [34].

In this study the proliferative and apoptotic activity in intact human colorectal epithelium from children and adults compared to that in adenoma and colorectal cancer was investigated. Expression of the related genes was also tested in mRNA microarrays. We found an increased proliferative and a decreased apoptotic activity in children and cancer samples compared to normal adult epithelium.

These results suggest an opposing molecular regulation of proliferation and apoptosis during normal aging and colorectal carcinogenesis. Enhanced cellular proliferation of tumor cells without “aging” can contribute to their survival advantage over adjacent senescent cells.

An increased cellular proliferation detected in children colorectal mucosa can be related to the physiologic growth of the large bowel however, the cell renewal slows down during normal aging in the histologically intact adult colonic crypt. Cell proliferation and apoptosis regulation between controlled growth in childhood and uncontrolled growth in CRC have not been correlated before in the colonic mucosa. Here we found several proliferation promoting genes including cyclin B1 /CCNB1/, cyclin E1 /CCNE1/ and cyclin-dependent kinase (CDK1) to be upregulated both in young and cancer samples compared to normal adult mucosa (*Figure 3*). These results correlate well with our finding significantly higher proliferating cell fractions in children and cancer tissue sections compared to normal adult samples. CDKs, indeed, have crucial role in the regulation of cell-cycle and growth in eukaryotic cells. CDK complexes are a highly conserved family of Ser/Thr protein kinases, consisting of a catalytic CDK subunit and an activating cyclin subunit. Different CDKs can control different parts of the cell-cycle; these complexes can be activated by phosphorylation, binding activating cyclins or inhibiting subunits [35]–[36]. CCNB1, CCNE1 and CDK1 expressions, which can correlate to an increased proliferative activity, were higher in children and neoplastic colonic mucosa compared to normal adult mucosa. Furthermore, cyclin D1 (CCND1), CDK1 and CDK6 mRNA levels were significantly higher in CRC compared to normal children samples, which may be related to the uncontrolled cellular proliferation in cancer (*Figure 4*). Cyclin D1 has several regulatory effects in normal cellular differentiation, growth and metabolism; however, the overexpression of CCND1 is one of the most commonly observed alteration in human cancers, as it has cell-cycle regulatory effects in G1 phase [37]. According to *Zhang et al.* results, the abnormal up-regulation of cyclin D1 can be an early event in intestinal carcinogenesis [38]. *Sahl et al.* have investigated the expression of different cyclin-dependent kinases in human colon cancer. They observed that the activation of CDK1, CDK2 and CDK6, which can phosphorylate the retinoblastoma protein, resulting in the release from the inhibition of forward progression of the G1 phase, is in connection with human colorectal carcinogenesis [39].

There are several regulatory molecules that can modulate and block the function of CKDs, called cyclin-dependent kinase inhibitors. In our present study, we investigated the mRNA expression alterations of CDKN2B in the processes of aging and colorectal carcinogenesis. CDKN2B is also known as multiple tumor suppressor 2 (MTS2), cyclin-dependent kinases 4 and 6 binding protein or p15-INK4b (p15). CDKN2B is located adjacent to tumor suppressor CDKN2A in the chromosome 9, which is frequently mutated and deleted in a wide variety of neoplasms. This gene encodes a cyclin-dependent kinase inhibitor that can produce complexes with CDK4 and CDK6, inhibiting the cellular growth or cell-cycle. In microarray analysis a moderate mRNA expression of CDKN2B was found in well-controlled, hyper-proliferative colonic biopsy samples from children as compared to histologically intact adult colonic mucosa, and a remarkable gene expression reduction was observed in CRC samples (*Figure 3*). According to previous studies, CDKN2B may act as a tumor suppressor gene and a potential effector of TGFβ-induced cell-cycle arrest [40]. *Herman et al.* certified that gene silencing of p15 by CpG island hypermethylation can cause neoplastic disorders [41]. Significant loss of these genes' functions may contribute to the uncontrolled cellular proliferation in CRC.

In immunohistochemical analysis, a moderately decreased apoptotic activity was found in healthy children colorectal biopsy samples compared to healthy adults and it was dramatically reduced in the course of adenoma-carcinoma sequence. mRNA expression alterations of apoptosis-inducing or -inhibiting genes analyzed in our study (e.g. ACVR1B, TNFSF10, DYRK2, SOCS3, IFI6 and SERPINB9) may explain this phenomenon. Since we observed a similarity of cellular proliferation and apoptosis tendency in healthy children and colorectal cancer, we tried to find a molecular discrepancy between the controlled and uncontrolled epithelial renewal.

At the mRNA level, significantly decreased somatostatin (SST) production was detected in CRC compared to that in healthy colonic biopsy samples from children; however, only a moderate decline in somatostatin expression in healthy adults was noted. SST is mainly secreted in the central nervous system; but its local secretion in the gastrointestinal tract is also well-documented. It has endocrine, paracrine effects; and through SST receptors, it directly exerts cell-cycle arrest and induces apoptosis [42]. After microarray analysis, we validated this observation on both dependent and independent sets of samples by using RT-PCR. According to our preliminary results we could presume that the local absence of SST production may contribute to the uncontrolled cellular proliferation in CRC.

We have analyzed and compared the proliferative and apoptotic activity in normal children, adult and CRC samples and tried to find the mRNA expression alterations in the background of controlled cellular proliferation in children and uncontrolled cellular proliferation in CRC. According to our immunohistochemical results, it has to be mentioned that cellular proliferation significantly decreases during physiological aging in histologically intact colorectal epithelium; moreover, the proliferative activity does not differ in normal adult and adenoma samples. However, the apoptotic activity is significantly lower in adenoma samples as compared that to normal adult samples. Thus, we can assume that a decreased apoptosis has a major role in the misbalanced cell-renewal and cell-death of the adenomatous status. In colorectal cancer, both of the increased cellular proliferation and nearly absent apoptosis may contribute to the uncontrolled cellular growth.

As we are aware, this is the first study to investigate mRNA expression in children colorectal biopsy samples with particular focus on proliferation and apoptosis regulation. Furthermore, these results were correlated with in situ mitosis and apoptosis index in comparison with those of normal adult samples and colorectal cancer. The lack of similar data prevented us to compare ours with other data sets on Gene Expression Omnibus databank. Since routine colonoscopy in children is rarely performed, collection from children colonic samples was the bottleneck of this study. In most children biopsies available for us other intestinal disorder e.g. inflammatory bowel disease, was diagnosed, which restricted our selection for only a few cases. This can explain both the lack of databank data and the relatively low number of such samples in our study.

In summary, we tested the proliferation- and apoptosis-related gene expression along with mitotic and apoptotic index in colorectal epithelium in the course of normal aging and colorectal carcinogenesis. We found similarly elevated proliferative and decreased apoptotic activities both in histologically intact children colonic mucosa and CRC samples compared to healthy adult samples, where cancer showed the significantly largest cell kinetic alterations. Physiological growth and development may result in an increased and controlled proliferative activity in children's colorectal epithelium which is a well-balanced process compared to the uncontrolled cellular proliferation in CRC. The distinct gene expression profiles between juvenile and cancer growth are likely to be regulated by genetic and epigenetic alterations.
